# Facilitators and Barriers to Implementing a Patient Portal at a Dental Hospital From the Implementers’ Perspectives: Qualitative Study

**DOI:** 10.2196/78979

**Published:** 2025-11-18

**Authors:** Adeola Bamgboje-Ayodele, Anna Paonne, Elise Evans, Michelle Ly, Allan So, Henna Solanki, Sonya So, Nicole Nixon, Adam Dunn, Martin Howell, Aaron Jones, Melissa Baysari

**Affiliations:** 1 Digital Health Human Factors Research Group, Sydney Nursing School, Faculty of Medicine and Health The University of Sydney New South Wales Australia; 2 Discipline of Design, School of Architecture, Design and Planning The University of Sydney New South Wales Australia; 3 Sydney Local Health District New South Wales Australia; 4 Five Faces Queensland Australia; 5 Biomedical Informatics and Digital Health, Faculty of Medicine and Health The University of Sydney Camperdown Australia; 6 Sydney School of Public Health, Menzies Centre for Health Policy, Faculty of Medicine and Health The University of Sydney Camperdown Australia

**Keywords:** patient portal, implementation, organization, barriers, facilitators, dental hospital

## Abstract

**Background:**

Patient portals can improve care delivery and optimize health service efficiency, but they are not always adopted by patients and staff. To improve adoption of patient portals, existing research has focused on examining implementation processes from the perspectives of end users. However, to our knowledge, the views of the implementers, who have in-depth insight into interorganizational and intraorganizational factors impacting implementation, have not been previously explored.

**Objective:**

The aim of this study was to explore implementers’ perspectives on the key facilitators and barriers to implementing a patient portal.

**Methods:**

This was a cross-sectional qualitative study conducted at a dental hospital in Sydney, New South Wales, Australia. Participants were implementers of a patient portal who were (1) administrative staff managers working at the dental hospital or (2) IT staff members who either worked within the district where the hospital is located or with the vendor. All implementers of the portal (N=13) took part in the interviews. Data were analyzed thematically and mapped to the Consolidated Framework for Implementation Research.

**Results:**

Eighteen factors were reported to affect the implementation of the patient portal: 7 (39%) acted as facilitators, 8 (44%) acted as barriers, and 3 (17%) factors both facilitated and hindered implementation. Many of the facilitators were related to the implementation process, such as planning and project management, leadership support, stakeholder motivation, and co-design. Most of the barriers occurred at the interorganizational level, such as delayed training for new staff, unique workflow and clinic characteristics, challenges with timeline and budget, and lack of direct vendor access to end users. While participants saw potential benefits of the portal for patients and the health service, they had concerns about increased staff workload related to the portal implementation. From the intraorganizational perspective, the lack of direct vendor access to end users was identified as a key barrier to implementation. From both interorganizational and intraorganizational perspectives, challenges with integrating the portal into the electronic medical record were identified as a persistent barrier to portal implementation.

**Conclusions:**

In addition to factors directly impacting clinicians and patients, our study highlighted that numerous organizational factors at multiple levels within and across organizations impacted successful patient portal implementation and adoption. Portal implementations would benefit from interoperability with existing systems, adequate resourcing, regular policy review related to technological change, and strategies to improve interagency communication among portal implementers. Our study offers learnings to health service organizations, implementers, and policymakers who aim to implement new portals or optimize existing patient portals.

## Introduction

As demand for health care services continues to rise, improving health service efficiency is a key priority for health care organizations. One way to do this is through the implementation of a patient portal [1-3]. A patient portal is a web-based application managed by health care organizations that supports patient appointment bookings, reminders, and communication with health care providers via secure messaging through the internet [4,5]. Research has shown that patient portals can improve health service efficiency by reducing missed appointments [6], the frequency of emergency department visits [7], and hospitalization [8].

Despite the potential benefits that patient portals can provide, evidence suggests that there is limited uptake of portals by patients in real-world settings [9]; for example, a meta-analysis of 40 studies on patient portal uptake showed a mean adoption rate of 23% in practice compared to 71% in controlled experiments [9]. Existing studies have also demonstrated mixed impacts on patients, health care providers, and health service organizations, likely because uptake of portals has been variable. Patient portals have been shown to improve patient engagement and satisfaction [10-12], but there remains conflicting evidence on the impact of patient portals on health service outcomes [13,14]. Current evaluation approaches tend to focus more on clinical end points and less on the complexity of the implementation in real-world settings, which could reveal insights into the factors impacting uptake [9].

Studies that focus on examining the implementation of patient portals, in particular identifying barriers and facilitators to the use of portals [15-18], typically focus on the perspectives of patients and staff, and there is limited research exploring the role of implementers and how they interacted with key stakeholders across implementation processes to enable portal delivery [19]. Implementers are those involved in designing and delivering strategies to ensure that new technologies or interventions are adopted and used effectively [20]. They typically include policymakers, IT professionals, or health professionals tasked with implementation [21]. A focus on implementers can uncover many interorganizational and intraorganizational factors impacting implementation that may not be visible or known to staff and patients. The aim of this study was to identify the perspectives of implementers on the key facilitators and barriers to implementing a patient portal.

## Methods

### Study Design

This was a cross-sectional qualitative study. As a qualitative study design is appropriate for providing insights into people’s experiences of a complex phenomenon or activity, we conducted semistructured interviews to explore the implementation team members’ experiences. The COREQ (Consolidated Criteria for Reporting Qualitative Research) checklist was used to report the study (Multimedia Appendix 1).

### Setting

This study was conducted at a public oral health service in Sydney, New South Wales, Australia. The oral health service has 160 dental chairs and sees on average 12,000 patients a month. A detailed description of the setting has been published elsewhere [4].

### Patient Portal Description

This study focused on a web-based application for managing patient appointments in outpatient clinics. The application, called Florence, was developed as an off-the-shelf solution by Five Faces. Florence comprises 2 components: a patient portal and a staff portal. More details on the portals are available in our previous publication, but the key functionalities are summarized in Table 1 [4]. Although Florence had been previously implemented in other clinics in the district where the hospital is located, it was introduced to this clinic to improve seamless communication between patients and administrative staff while providing an opportunity to reduce failure-to-attend rates [4,22]. Florence went live at one clinic at the dental hospital on December 6, 2023, targeting planned research to evaluate the implementation of the portal.

**Table 1:** Functionalities embedded in both the patient and staff portals.

Portal features	Patient portal	Staff portal
View upcoming appointments	✓	
Send messages to patients or receive messages from the clinic	✓	✓
Confirm upcoming appointments	✓	
Request appointment cancellation	✓	
Check in upon arrival to the clinic	✓	
Request appointment rescheduling	✓	
Call patients to reception or to a clinical room		✓

### Participants and Procedure

Using purposive sampling, all staff members who were involved in the implementation of Florence were invited by email to participate in interviews. Participants were identified by an implementation officer who was a member of the research team and supported the implementation team at the dental hospital. Participants were eligible for an interview if they took part in any pre- or postimplementation activity related to Florence and were (1) administrative staff managers working at the dental hospital and involved in activities such as implementation planning, monitoring, and reporting; or (2) IT staff members (eg, change managers, project managers, or data analysts) who either worked within the local health district where the hospital is located or with the vendor (ie, Five Faces) and were involved in activities such as technical preparation, training, communication, testing, and support. All participants who were invited to an interview agreed to take part.

Semistructured interview questions (Multimedia Appendix 2) were developed for this study by research team members with expertise in technology implementation and evaluation, implementation science, and human factors (AS, MB, and AB-A) and were broadly guided by the Consolidated Framework for Implementation Research (CFIR), an implementation science framework guiding the successful implementation of innovations [23]. The CFIR includes 6 domains: intervention characteristics, inner setting, outer setting, individual characteristics, implementation process, and outcomes [23]. Participants were asked to describe their experiences implementing Florence and to reflect on aspects that worked well and those that did not. Although the study used purposive sampling and all eligible participants were invited to participate, data collection continued until thematic saturation was achieved. In this case, saturation coincided with the participation of all invited individuals. All interviews were conducted online, recorded, and transcribed verbatim. The interviews were conducted by junior researchers (AS, EE, and ML) who were trained in conducting interviews by a professor of human factors (MB). The interviewers had no prior relationship with the participants.

### Data Analysis and Interpretation

Our analytical approach included familiarizing ourselves with the data, coding, generating initial themes, reviewing potential themes, defining and naming themes, and producing the report [24]. Deidentified transcripts were thematically analyzed manually and independently by EE and ML, who first identified and coded the transcribed data through inductive thematic analysis, using a data-driven approach to understand participants’ experiences and perceptions [25,26]. After generating the initial themes, EE and ML held meetings with AB-A to review each line of code, discuss discrepancies, and generate potential themes. Subsequently, AB-A iteratively held meetings with EE and ML to refine the themes and codes until a consensus was reached. Guided by the CFIR framework, the researchers conducted a 2-hour workshop to map the identified themes to 1 of the 6 domains of the CFIR via a deductive analysis process.

### Ethical Considerations

This study was reviewed and approved by the Sydney local health district’s human research ethics committee (X23-0274 and 2023/ETH01612). All appropriate ethical safeguards were implemented to ensure the anonymity and confidentiality of participants. Before participation, individuals were fully informed of the study’s aims and procedures. They were also made aware of how their data would be used, including assurances that all responses would be anonymized and treated with strict confidentiality. Consent was obtained from all participants, and they were informed of their right to withdraw from the study at any time. Participation was voluntary. No reimbursement was provided. No identifiable data are included in this manuscript or the supplementary files.

## Results

### Overview

Individual semistructured interviews were conducted with all 13 invited participants and lasted 19 minutes on average (SD 5.6, 243 min in total). Participants were generally balanced by sex assigned at birth, were all full-time staff, and had a range of experience. Their ages ranged from 34 to 66 years (Table 2).

**Figure 1:**
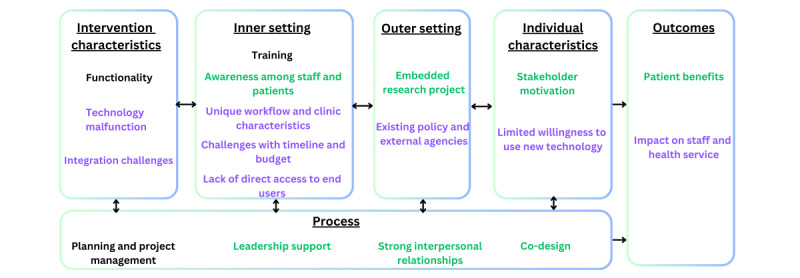
Themes mapped to the Consolidated Framework for Implementation Research. Green indicates facilitator or advantage (in outcomes), purple indicates barrier or disadvantage (in outcomes), and black indicates both barrier and facilitator.

Our findings revealed 18 themes that were mapped to the 6 domains of the CFIR framework. Of these 18 themes, 7 (39%) were facilitators, and 8 (44%) were barriers, while 3 (17%) had dual traits (ie, emerged as both a facilitator and a barrier; Figure 1 and Textbox 1).

**Table 2:** Demographic characteristics of participants (N=13).

Demographic characteristics		Values
Age (years), mean (SD; range)		46 (9.8; 34-66)
**Sex assigned at birth, n (%)**		
	Female	7 (54)
	Male	6 (46)
**Employment type, n (%)**		
	Full time	13 (100)
**Work experience (years), n (%)**		
	5-9	1 (8)
	10-14	4 (31)
	15-19	2 (15)
	≥20	6 (46)
**Organization, n (%)**		
	Vendor	2 (15)
	IT staff	9 (69)
	Administrative staff at dental hospital	2 (15)

**List of facilitators and barriers to implementing the patient portal.**

Facilitator (or advantage)

- Awareness among staff and patients
- Embedded research project
- Stakeholder motivation
- Leadership support
- Strong interpersonal relationships
- Co-design
- Patient benefits

Barrier (or disadvantage)

- Technology malfunction
- Integration challenges
- Unique workflow and clinic characteristics
- Challenges with timeline and budget
- Lack of direct access to end users
- Existing policy and external agencies
- Limited willingness to use new technology
- Impact on staff and health service

Both barrier and facilitator

- Functionality
- Training
- Planning and project management

### Intervention Characteristics

Three themes emerged in the intervention characteristics domain: functionality, technology malfunction, and integration challenges.

#### Functionality

Participants described how the functionalities of the portal were viewed as both a facilitator and a barrier to implementation. As a facilitator, participants reported that the ability of the portal to provide visibility of appointment lists was helpful, with a participant stating that “people [patients] appreciated having that digital resource that they can see their appointment list that they’ve got visibility” (P004). As a barrier, participants noted that the portal lacked a task delegation functionality for staff, which negatively impacted administrative workflow. One participant stated as follows:

I think, an issue around as well, there being quite a big admin team that is working at the clinic, and they’re not all sat in the same place. So there’s just difficulties around knowing who is responding to messages.

P010

#### Technology Malfunction

As a barrier to implementation, participants reported instances where the technology malfunctioned, which disrupted workflow and resulted in patient confusion:

There was an issue when the patients were getting the messages after being checked in on to their mobile phone. So even after checking in, the patients were coming to the desk, “I got a message on my phone, do you need to see me again?”

P005

#### Integration Challenges

Many participants described challenges with integrating the portal with the electronic medical record (EMR) as a barrier to implementation. One participant explained how the complexity of EMR integration impacted their implementation timeline:

I think there are a few complexities based on our other implementations. Just because we’re using [the EMR], which we hadn’t used before.... So I think it was just to probably a bit more time in discovery and writing requirements because we were sort of had to do that groundwork from the beginning again.... So I think if anything, it just took a little bit longer than normal.

P004

### Inner Setting

Five themes emerged in this domain: training, awareness among staff and patients, unique workflow and clinic characteristics, challenges with timeline and budget, and lack of direct access to end users.

#### Training

Participants reported training as both a facilitator and a barrier to implementation. As a facilitator, they noted that patient-facing administrative staff received adequate training on the portal:

I think those [training] sessions worked really well, giving everybody that overall view of what Florence is.

P006

In addition, many participants mentioned the availability of resources, such as quick reference guides, to support their use of the portal*:*

Every one of us [district staff] has got the training and they [IT staff] have left the paperwork as well in case we are stuck…we know what people to refer to…that was good.

P005

However, some other participants noted that it was challenging for new staff members to successfully use the tool because organizing Florence training for new staff was time consuming:

It does pose a challenge when someone hasn’t been trained or told about Florence and they’ve rotated to the place and suddenly there’s a quick rushed handover.

P007

#### Awareness Among Staff and Patients

Participants viewed awareness among administrative staff and patients as a facilitator of successful portal implementation, and this was reported to result in the portal being accepted; for example, a participant reported that patients were adequately informed before the implementation of the portal:

They were informing patients, “hey, this is coming”...so just giving that little bit of heads up of the change...that’s probably why it was received well.

P009

#### Unique Workflow and Clinic Characteristics

Participants reported that the unique workflow and characteristics of the clinic were barriers to implementation. They further noted that the workflow was unfamiliar to the IT staff, which resulted in an increase in the time needed to understand it:

And I think the way that the clinics run there are also quite different to a lot of the outpatient clinics within the other facilities. So that’s presented some workflow-related issues...[to] resolve some of those issues probably [took] a bit longer than what we would typically see at the other clinics in the district.

P010

#### Challenges With Timeline and Budget

Participants described how timeline and budget constraints impacted the quality of the portal that was implemented:

It’s always a compromise in software, so you have to always come up with things to finish on time or on budget, but still, you kind of feel like you didn’t do the best job that you could have.

P011

It was reported that difficulty in meeting timelines arose from budgetary constraints:

There were some things left to the very last minute and very tight timelines because we couldn’t get budgets approved through [the district].

P001

#### Lack of Direct Access to End Users

Participants reported that the vendor’s lack of direct access to staff and patients was a barrier that resulted in limited opportunities for co-design and iterative development of the portal:

I think you know, because we don’t have that direct contact with [the dental] hospital and we’re going by the IT team [in the district], it’s their project and their solution.

P001

### Outer Setting

Two themes were identified in this domain: the embedded research project and external policy and external agencies.

#### Embedded Research Project

Aligning portal implementation with the research project was viewed as a facilitator. Participants noted that the research timeline facilitated rapid approval processes within the district:

I think having this project as a research project kind of was able to influence that approval process a little bit faster than it would normally...so that was well done.

P001

#### Existing Policy and External Agencies

Participants described the existing policy that required administrative staff members to physically sight patients’ Medicare or concession cards at every visit as a barrier to portal implementation. This policy prevented the portal’s kiosk from being used, which could have allowed patients to check in digitally at each visit. Other participants stressed that the policy negatively impacted staff workflow, resulting in inefficiencies:

Every patient needs to present to reception, it’s part of the workflow. They need to have their concession card and Medicare card sighted physically. So there’s no real saving of time by having certain patients be able to just sit down and see clinicians directly.

P008

In addition, participants stated that it was difficult to engage with other government agencies involved in providing relevant infrastructure. They explained that the government agency involved in the implementation did not provide relevant staff with updates on the progress of database extraction:

The only issue would have been that, since the databases are owned by [government agency], they were the ones who would have to set up the database extraction...We request and we have to do the following up. They don’t tell us, either they can’t do it now or they have done it.

P012

### Individual Characteristics

Two themes emerged in this domain: stakeholder motivation and limited willingness to use new technology.

#### Stakeholder Motivation

Participants reported that they were motivated to support the implementation of the portal; for example, a participant described being driven by the portal’s potential benefits for patients:

I’m gonna use the word proud that we’ve implemented this solution at [the dental] hospital.... Yes, I can talk about the delivery and so forth, but it’s about what the benefit is gonna be to our consumers...those were important to me.

P003

Another participant reported that they were motivated by the stakeholders at the dental hospital:

I think some of the stakeholders at [the dental] hospital really worked well with us to get across the line, hav[ing] them on board and motivated for this change was imperative for success.

P006

#### Limited Willingness to Use New Technology

A major barrier to successful portal implementation was resistance to the technology among both patient and staff; for example, participants attributed negative comments from patients about the portal’s usability to their lack of willingness to use it:

We’ve had a few thumbs down, which are comments that you know are fairly relevant. “I’m too old,” “how to use it,” or “I couldn’t get the OTP code.”

P004

Administrative staff were also viewed as being set in their ways of working and unwilling to change:

I think that these guys [administrative staff] in particular may have been very set in processes that they have been doing for a very long time.

P006

### Implementation Process

Four themes emerged in the implementation process domain: planning and project management, leadership support, strong interpersonal relationships, and co-design.

#### Planning and Project Management

Participants described the process of planning and project management as both a facilitator and a barrier to implementation. Good planning and project management were reported to enable successful implementation:

We have our own internal team boards and that’s how we track everything...once a week we have a long team stand-up...so that really helps keep on track.

P004

However, delays in receiving approvals from the district were reported to be a barrier to the successful implementation of Florence. One participant explained that the delays impacted their ability to remember the requirements or the context of the situation:

And because [requirements to be approved] took a long time, by the time we were approved, I would go back to the documentation and just not remember or struggle to remember that context of “why did I write that” or “what’s the context of this requirement in terms of workflow.”

P008

#### Leadership Support

Participants identified leadership support as critical to the successful implementation of Florence and stressed the importance of the leader’s capabilities, collaborative nature, and management skills:

I think when [staff name; leadership position] came on board, things came together, and she was able to make decisions.

P004

Another participant highlighted that an individual in a leadership position provided significant support to drive the success of the implementation among administrative staff members:

The admin supervisor, she’s been amazing to work with...really forthcoming with coordinating our team...sort of connecting us with other things...it seems like she’s got a very good relationship with the admin staff there as well, which means that they can give their honest feedback.

P010

#### Strong Interpersonal Relationships

Participants revealed that vendor and district stakeholders had strong interpersonal relationships, which facilitated collaboration and transparency:

I would say maybe a lot of it is we have a relationship already with the LHD [local health district], so we have been working together for a while. They know how we understand things, we know how they understand things.

P011

Another participant recalled how “there’s full transparency between what we’re doing and what they need...we just have full transparency and a really great relationship” (P001).

#### Co-Design

Although limited, co-design opportunities were viewed as a key facilitator, allowing the vendor to understand existing workflows and improve portal functionalities:

We did a lot of workshops...trying to really understand the workflows and understanding the technical side of [the EMR] as well.... I think unless we can really see it for ourselves, it’s very hard to test and know what’s going to happen.... I think that [we] really benefited having that access.

P004

In addition, multiple participants from both the hospital and the vendor stressed the importance of on-site visits in guiding decision-making, communication, and team building:

I think with a combination of having team calls, but then also being able to visit on site, I think both is really important, and we were able to do both quite easily.

P010

### Outcomes

Staff reported 2 advantages and 1 disadvantage of the portal. These were related to patient benefits and the impact on staff and the health service.

#### Patient Benefits

Participants reported that the portal helped patients manage their appointments by enabling online communication with the clinic:

In terms of putting the appointment into patient’s hands so that they can manage it, I think that’s a big win for them.

P013

The portal was also reported to be beneficial for supporting asynchronous communication:

It’s almost like patients feel more comfortable talking through text...they’re more elaborate [in] explaining their experience.

P009

#### Impact on Staff and Health Service

It was noted that staff benefited from the portal, as it reduced the time spent on certain tasks; for example, a participant described how it simplified confirming patient appointments:

It was 120 appointments confirmed and I sort of sat there and I’m like “my goodness, if we tried to call 120 patients, that’s the whole day’s work,” so I think the efficiencies [of the portal] were almost immediately realized.

P007

In addition, participants noted that that portal had “address[ed] things like no-shows” (P008).

However, participants also reported that the portal increased staff workload. Specifically, they noted that the existing policy requiring administrative staff to physically sight patients’ Medicare or concession cards at each visit was a barrier to implementation. This policy complicated the check-in process, as patients had to queue at both the Florence kiosk and the reception desk, reportedly increasing administrative staff workload:

I would rather have that patient come into the desk anyways...because we have to check the Medicare cards and the Centrelink cards physically to sight them before arriving the patient through Florence. So that’s an added step for the admin officers.

P005

It [checking patients in manually] gets very overwhelming, especially when we are returning from our breaks, we physically have to check in every patient and call out every patient. The patients also need to be called out to the reception because they are not aware.

P005

## Discussion

### Principal Findings

In this study, we sought to understand the barriers and facilitators to implementing a patient portal from the perspectives of those involved in the implementation. We identified 18 factors impacting implementation: 7 (39%) acted as facilitators, 8 (44%) acted as barriers, and 3 (17%) both facilitated and hindered implementation. Many of the facilitators were related to the implementation process, such as planning and project management, leadership support, stakeholder motivation, and co-design. Most of the barriers occurred at the interorganizational level, such as delayed training for new staff, unique workflow and clinic characteristics, challenges with timeline and budget, and lack of direct vendor access to end users. While participants saw potential benefits of the portal for patients and the health service, increased staff workload as a result of portal implementation was flagged as a concern.

From an interorganizational perspective, other studies have highlighted the importance of support from government agencies and appropriate policies to drive patient engagement, portal adoption, and successful implementation [17]; for example, the “meaningful use” requirements of the Medicare and Medicaid Electronic Health Record Incentive programs has largely driven the development and implementation of patient portals in the United States [27,28]. Moreover, in Sweden, the government supported the implementation of the national patient portal to deliver successful digital health innovations [29]. Our study sheds light on the relationship between government agencies and the implementation team, with implementers reporting difficulty in engaging with government agencies to support the technical aspects of portal implementation. Effective communication between internal and external agencies was previously identified as necessary for successful health IT implementation during the COVID-19 pandemic [30].

Our study adds to the body of knowledge highlighting the importance of effective interagency communication and strategies such as improved transparency and responsiveness [30] that support communication between government agencies, health service organizations, and vendors.

A policy that was in place before portal implementation emerged as a barrier to the use of the portal’s kiosk in this study. This is consistent with a mixed methods study that assessed the impact of implementing a multi-institutional patient portal on stakeholder adoption and use and identified the need for policy support to streamline implementation efforts [19]. In the study, the authors flagged the importance of ensuring that policies consider factors that enable data sharing, such as age and capacity to consent, as well as provisions for proxy access to delegate users. In our study, policy prevented the assessment of electronic concession cards during the check-in process and mandated a somewhat antiquated process of physically sighting a patient’s physical card. Given the fast pace at which technology is moving (eg, blockchain technology can now be used to store and control access to digital wallets containing patient data) [31,32] and the fact that many patients now prefer using digital wallets via their smartphones to physical wallets [33], health services and government need to be proactive and future focused and ensure that policies do not lag behind or restrict technology innovation [22].

When considering the intraorganizational perspective, our research also revealed the lack of direct vendor access to end users as a barrier to implementation. This finding differs from other research, as many studies have documented the use of a participatory or co-design approach involving end users to develop patient portals [34,35]. One reason for this difference is that our research captured the vendor perspective, highlighting the need for better access to end users without creating a “middleman problem,” a view that was not explicitly captured in previous studies [34,35]. A middleman problem is a situation in which IT staff, who should serve as a bridge between health care providers and vendors, act as gatekeepers (ie, those who control access and filter information between both parties) [36]. The middleman problem is a significant challenge, as user requirements can become lost in translation between the end users and the vendor [36]. We have previously called for health services to provide protected time for health care providers to participate in co-design of health IT and for vendors to provide clarity on the expectation of time commitment [36]. Our findings further support this.

Leadership support and stakeholder motivation were identified as key enablers of portal implementation. This is consistent with findings from existing literature showing that leadership support is essential for fostering the uptake and implementation of patient portals [19]. A scoping review of 10 systematic reviews on patient portals revealed that leadership support drove adoption through efforts such as working with developers on portal design, developing policies for user training, and integrating portals into staff workflows [37]. The review also identified patient and staff engagement as a key facilitator of portal uptake [37]. To ensure end-user awareness (identified as an enabler in our inner setting domain), stakeholder engagement strategies, such as the advertising prepared for patients, carers, and staff, must be tailored to the targeted population groups and the local context to be effective [37].

From both interorganizational and intraorganizational perspectives, we found challenges with EMR integration to be a key barrier, consistent with previous research [19]. Without interoperable systems, the expected benefits of patient portals through secure and efficient exchange of patient data may not be realized, and this could pose risks to care quality and health system efficiency [38]; for example, we found that interoperability issues between the portal and the EMR were perceived to increase workload for administrative staff. Consequently, ensuring interoperability across relevant systems could minimize or mitigate additional workload associated with portal implementation. A scoping review of 29 patient portals identified interoperability between patient portals and electronic health records as one of the key requirements [39]. The review revealed that enablers of interoperability extend beyond technical solutions to include organizational factors (eg, stakeholder engagement) and collaboration with relevant agencies to develop consistent regulations and standards [39].

### Strengths and Limitations of the Study

Our study used the CFIR framework to examine the facilitators and barriers to implementing a patient portal at a dental hospital from the implementers’ perspectives. Similar studies have focused on the perspectives of end users, but our study was unique in that it explored the perspectives of the implementers, a view typically absent in the literature. Our findings are based on data collected via interviews conducted within a single health care organization, which may limit generalizability to other contexts and interventions. We also acknowledge that the patient-centered and staff views highlighted in our study were those reported by the implementers and not patients or the staff themselves, but exploring patient experiences with the portal was a complementary component of this research and is reported elsewhere [22]. The majority of the participants (9/13, 69%) in this study were IT staff, whose perspectives were shaped by their technical roles and responsibilities in the implementation of Florence. While their insights provided valuable depth regarding system functionality, integration, and operational challenges, the limited representation from administrative and vendor staff may have constrained the breadth of perspectives captured. Greater participation from these groups could have yielded additional insights. Future research may benefit from a more balanced representation across stakeholder groups to enrich understanding of implementation processes. While our study focused on actual portal implementers, future studies could include perspectives from clinics planning to adopt the portal to provide valuable insights into anticipated barriers and facilitators.

### Conclusions

Our findings are being used to inform the subsequent implementation of the portal across all other clinics within the dental hospital. In addition to factors directly impacting clinicians and patients, our study highlighted that a large number of organizational factors at multiple levels within and across organizations impacted successful patient portal implementation and adoption. Our findings suggest that successful implementation of patient portals requires (1) interoperability with existing systems, (2) adequate resources (including protected time for health care providers to participate in co-design), (3) review of existing policies to enable technology alignment with workflow, and (4) strategies from government agencies to improve interagency communication with portal implementers.

## Data Availability

The datasets generated and analyzed during this study are not publicly available due to ethical restrictions but are available from the corresponding author on reasonable request.

## Appendix

Multimedia Appendix 1 COREQ checklist.

Multimedia Appendix 2 Interview guide.

## Abbreviations

CFIR: Consolidated Framework for Implementation Research
COREQ: Consolidated Criteria for Reporting Qualitative Research
EMR: electronic medical record
