# Supplementary material for: Facilitators and Barriers to Implementing a Patient Portal at a Dental Hospital From the Implementers’ Perspectives: Qualitative Study
Source: J Med Internet Res. 2025 Nov 18;27:e78979. doi: 10.2196/78979 (PMC12673303; doi:10.2196/78979)
Supplement: Multimedia Appendix 1 [file jmir_v27i1e78979_app1.pdf]

COREQ Checklist

Topic	Item No.	Guide Questions	Page No.
**Domain 1: Research team and reflexivity**			
Personal Characteristics			
Interviewer/facilitator	1	Which author/s conducted the interview or focus group?	5
Credentials	2	What were the researcher's credentials? *e.g. PhD, MD*	-
Occupation	3	What was their occupation at the time of the study?	5
Gender	4	Was the researcher male or female?	N/A
Experience and training	5	What experience or training did the researcher have?	5
Relationship with participants			
Relationship established	6	Was a relationship established prior to study commencement?	5
Participant knowledge of the interviewer	7	What did the participants know about the researcher? *e.g. personal goals, reasons for doing the research*	Appendix B
Interviewer characteristics	8	What characteristics were reported about the interviewer/facilitator? *e.g. Bias, assumptions, reasons and interests in the research topic*	Appendix B
**Domain 2: Study design**			
Theoretical framework			
Methodological orientation and Theory	9	What methodological orientation was stated to underpin the study? e.g. *grounded theory, discourse analysis, ethnography, phenomenology, content analysis*	6
Participant selection			

Sampling	10	How were participants selected? *e.g. purposive, convenience, consecutive, snowball*	5
Method of approach	11	How were participants approached? *e.g. face-to-face, telephone, mail, email*	5
Sample size	12	How many participants were in the study?	6
Non-participation	13	How many people refused to participate or dropped out? Reasons?	N/A
Setting			
Setting of data collection	14	Where was the data collected? *e.g. home, clinic, workplace*	5
Presence of non-participants	15	Was anyone else present besides the participants and researchers?	N/A
Description of sample	16	What are the important characteristics of the sample? *e.g. demographic data*	6<br>Table 1
Data collection			
Interview guide	17	Were questions, prompts, guides provided by the authors? Was it pilot tested?	Appendix A
Repeat interviews	18	Were repeat interviews carried out? If yes, how many?	N/A
Audio/visual recording	19	Did the research use audio or visual recording to collect the data?	5
Field notes	20	Were field notes made during and/or after the interview or focus group?	N/A
Duration	21	What was the duration of the interviews or focus group?	6
Data saturation	22	Was data saturation discussed?	5
Transcripts returned	23	Were transcripts returned to participants for comment and/or correction?	N/A
**Domain3: Analysis and findings**			
Data analysis			

Number of data coders	24	How many data coders coded the data?	6
Description of the coding tree	25	Did authors provide a description of the coding tree?	7
Derivation of themes	26	Were themes identified in advance or derived from the data?	6
Software	27	What software, if applicable, was used to manage the data?	N/A
Participant checking	28	Did participants provide feedback on the findings?	N/A
Reporting			
Quotations presented	29	Were participant quotations presented to illustrate the themes/findings? Was each quotation identified? *e.g. participant number*	6-15
Data and findings consistent	30	Was there consistency between the data presented and the findings?	6-15
Clarity of major themes	31	Were major themes clearly presented in the findings?	6-15
Clarity of minor themes	32	Is there a description of diverse cases or discussion of minor themes?	15-19
