# Supplementary material for: Facilitators and Barriers to Implementing a Patient Portal at a Dental Hospital From the Implementers’ Perspectives: Qualitative Study
Source: J Med Internet Res. 2025 Nov 18;27:e78979. doi: 10.2196/78979 (PMC12673303; doi:10.2196/78979)
Supplement: Multimedia Appendix 2 [file jmir_v27i1e78979_app2.docx]

**Semi-structured Interview Questions**

**Interview Structure**

Before the interview starts, are you happy for me to record our interview and use it for research purposes? *PROMPT ANSWER*.

First of all, I would like to thank you for agreeing to be interviewed to allow us to better understand the implementation of Florence at COHC. The interview is confidential and the answers provided will be used to let us understand how we can improve implementation of digital health platforms in the future. As mentioned, the interview will be recorded for the sake of the recording can you please confirm this is okay.

We are currently undertaking internships at the University of Sydney and are here to help gain the implementer’s perspective (you) to inform the next phase of Florence’s research. Keep in mind I will be taking notes on the side and this is just to keep track of your responses, so if I am taking notes please feel free to continue your response.

Okay, fantastic! Let's get into the interview questions.

**Interview Questions**

1. So can you please tell me what your official title is and what part you played in the implementation of Florence?
2. How have you found the implementation of Florence at COHC so far?
3. What has gone well?
4. What hasn’t gone well?
   1. How did this impact you?
   2. What did you do to address some of these issues?
5. What are some of the barriers you encountered during the implementation?
   1. Organisation level?
   2. External Barriers?
   3. Barriers relating to people?
   4. Technological barriers?
6. If you had to do it again, would you have done anything differently?
7. Do you think Florence in its current form meets the needs of the COHC? How and why?
8. During the implementation of Florence, how did you engage with stakeholders and users before, during and after the Implementation of Florence?
